# Supplementary material for: Glycosylphosphatidylinositol biosynthesis and remodeling are required for neural tube closure, heart development, and cranial neural crest cell survival
Source: eLife. 2019 Jun 24;8:e45248. doi: 10.7554/eLife.45248 (PMC6611694; doi:10.7554/eLife.45248)
Supplement: Supplementary file 1. [file elife-45248-supp1.pptx]

## Slide 1
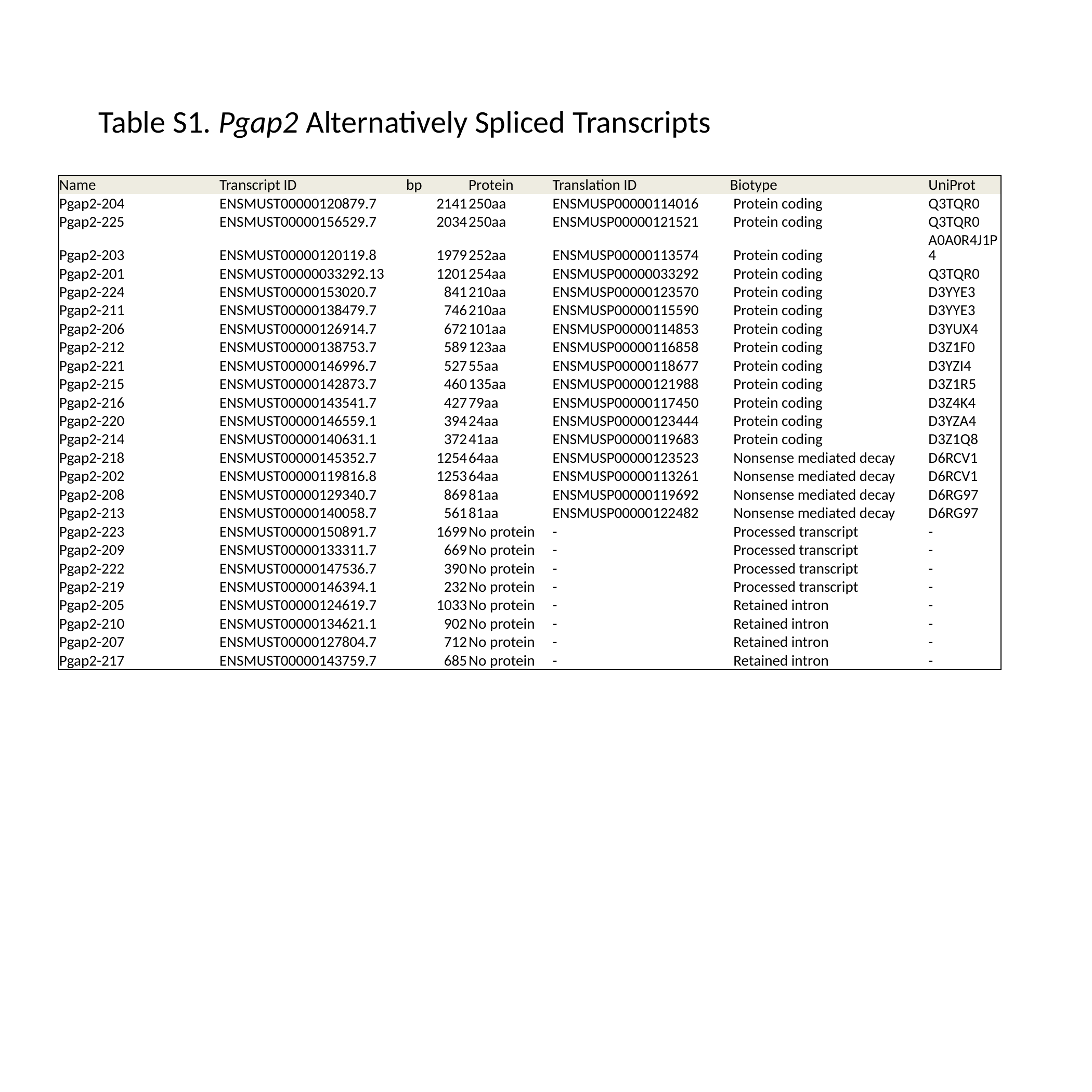

Table S1. Pgap2 Alternatively Spliced Transcripts
| Name | Transcript ID | bp | Protein | Translation ID | Biotype | UniProt |
| --- | --- | --- | --- | --- | --- | --- |
| Pgap2-204 | ENSMUST00000120879.7 | 2141 | 250aa | ENSMUSP00000114016 | Protein coding | Q3TQR0 |
| Pgap2-225 | ENSMUST00000156529.7 | 2034 | 250aa | ENSMUSP00000121521 | Protein coding | Q3TQR0 |
| Pgap2-203 | ENSMUST00000120119.8 | 1979 | 252aa | ENSMUSP00000113574 | Protein coding | A0A0R4J1P4 |
| Pgap2-201 | ENSMUST00000033292.13 | 1201 | 254aa | ENSMUSP00000033292 | Protein coding | Q3TQR0 |
| Pgap2-224 | ENSMUST00000153020.7 | 841 | 210aa | ENSMUSP00000123570 | Protein coding | D3YYE3 |
| Pgap2-211 | ENSMUST00000138479.7 | 746 | 210aa | ENSMUSP00000115590 | Protein coding | D3YYE3 |
| Pgap2-206 | ENSMUST00000126914.7 | 672 | 101aa | ENSMUSP00000114853 | Protein coding | D3YUX4 |
| Pgap2-212 | ENSMUST00000138753.7 | 589 | 123aa | ENSMUSP00000116858 | Protein coding | D3Z1F0 |
| Pgap2-221 | ENSMUST00000146996.7 | 527 | 55aa | ENSMUSP00000118677 | Protein coding | D3YZI4 |
| Pgap2-215 | ENSMUST00000142873.7 | 460 | 135aa | ENSMUSP00000121988 | Protein coding | D3Z1R5 |
| Pgap2-216 | ENSMUST00000143541.7 | 427 | 79aa | ENSMUSP00000117450 | Protein coding | D3Z4K4 |
| Pgap2-220 | ENSMUST00000146559.1 | 394 | 24aa | ENSMUSP00000123444 | Protein coding | D3YZA4 |
| Pgap2-214 | ENSMUST00000140631.1 | 372 | 41aa | ENSMUSP00000119683 | Protein coding | D3Z1Q8 |
| Pgap2-218 | ENSMUST00000145352.7 | 1254 | 64aa | ENSMUSP00000123523 | Nonsense mediated decay | D6RCV1 |
| Pgap2-202 | ENSMUST00000119816.8 | 1253 | 64aa | ENSMUSP00000113261 | Nonsense mediated decay | D6RCV1 |
| Pgap2-208 | ENSMUST00000129340.7 | 869 | 81aa | ENSMUSP00000119692 | Nonsense mediated decay | D6RG97 |
| Pgap2-213 | ENSMUST00000140058.7 | 561 | 81aa | ENSMUSP00000122482 | Nonsense mediated decay | D6RG97 |
| Pgap2-223 | ENSMUST00000150891.7 | 1699 | No protein | - | Processed transcript | - |
| Pgap2-209 | ENSMUST00000133311.7 | 669 | No protein | - | Processed transcript | - |
| Pgap2-222 | ENSMUST00000147536.7 | 390 | No protein | - | Processed transcript | - |
| Pgap2-219 | ENSMUST00000146394.1 | 232 | No protein | - | Processed transcript | - |
| Pgap2-205 | ENSMUST00000124619.7 | 1033 | No protein | - | Retained intron | - |
| Pgap2-210 | ENSMUST00000134621.1 | 902 | No protein | - | Retained intron | - |
| Pgap2-207 | ENSMUST00000127804.7 | 712 | No protein | - | Retained intron | - |
| Pgap2-217 | ENSMUST00000143759.7 | 685 | No protein | - | Retained intron | - |
