# Supplementary material for: Glycosylphosphatidylinositol biosynthesis and remodeling are required for neural tube closure, heart development, and cranial neural crest cell survival
Source: eLife. 2019 Jun 24;8:e45248. doi: 10.7554/eLife.45248 (PMC6611694; doi:10.7554/eLife.45248)
Supplement: Supplementary file 2. [file elife-45248-supp2.pptx]

## Slide 1
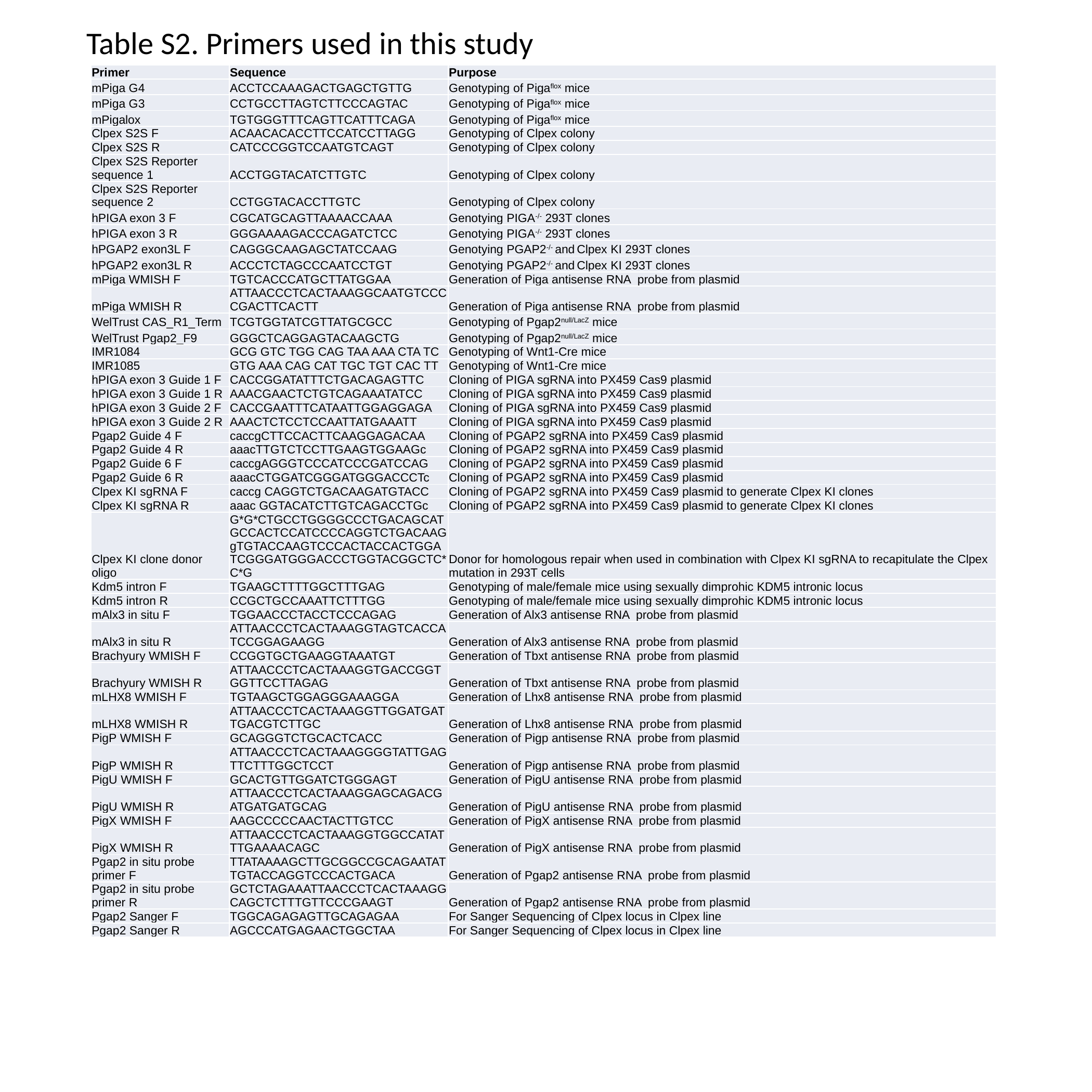

Table S2. Primers used in this study
| Primer | Sequence | Purpose |
| --- | --- | --- |
| mPiga G4 | ACCTCCAAAGACTGAGCTGTTG | Genotyping of Pigaflox mice |
| mPiga G3 | CCTGCCTTAGTCTTCCCAGTAC | Genotyping of Pigaflox mice |
| mPigalox | TGTGGGTTTCAGTTCATTTCAGA | Genotyping of Pigaflox mice |
| Clpex S2S F | ACAACACACCTTCCATCCTTAGG | Genotyping of Clpex colony |
| Clpex S2S R | CATCCCGGTCCAATGTCAGT | Genotyping of Clpex colony |
| Clpex S2S Reporter sequence 1 | ACCTGGTACATCTTGTC | Genotyping of Clpex colony |
| Clpex S2S Reporter sequence 2 | CCTGGTACACCTTGTC | Genotyping of Clpex colony |
| hPIGA exon 3 F | CGCATGCAGTTAAAACCAAA | Genotying PIGA-/- 293T clones |
| hPIGA exon 3 R | GGGAAAAGACCCAGATCTCC | Genotying PIGA-/- 293T clones |
| hPGAP2 exon3L F | CAGGGCAAGAGCTATCCAAG | Genotying PGAP2-/- and Clpex KI 293T clones |
| hPGAP2 exon3L R | ACCCTCTAGCCCAATCCTGT | Genotying PGAP2-/- and Clpex KI 293T clones |
| mPiga WMISH F | TGTCACCCATGCTTATGGAA | Generation of Piga antisense RNA probe from plasmid |
| mPiga WMISH R | ATTAACCCTCACTAAAGGCAATGTCCCCGACTTCACTT | Generation of Piga antisense RNA probe from plasmid |
| WelTrust CAS\_R1\_Term | TCGTGGTATCGTTATGCGCC | Genotyping of Pgap2null/LacZ mice |
| WelTrust Pgap2\_F9 | GGGCTCAGGAGTACAAGCTG | Genotyping of Pgap2null/LacZ mice |
| IMR1084 | GCG GTC TGG CAG TAA AAA CTA TC | Genotyping of Wnt1-Cre mice |
| IMR1085 | GTG AAA CAG CAT TGC TGT CAC TT | Genotyping of Wnt1-Cre mice |
| hPIGA exon 3 Guide 1 F | CACCGGATATTTCTGACAGAGTTC | Cloning of PIGA sgRNA into PX459 Cas9 plasmid |
| hPIGA exon 3 Guide 1 R | AAACGAACTCTGTCAGAAATATCC | Cloning of PIGA sgRNA into PX459 Cas9 plasmid |
| hPIGA exon 3 Guide 2 F | CACCGAATTTCATAATTGGAGGAGA | Cloning of PIGA sgRNA into PX459 Cas9 plasmid |
| hPIGA exon 3 Guide 2 R | AAACTCTCCTCCAATTATGAAATT | Cloning of PIGA sgRNA into PX459 Cas9 plasmid |
| Pgap2 Guide 4 F | caccgCTTCCACTTCAAGGAGACAA | Cloning of PGAP2 sgRNA into PX459 Cas9 plasmid |
| Pgap2 Guide 4 R | aaacTTGTCTCCTTGAAGTGGAAGc | Cloning of PGAP2 sgRNA into PX459 Cas9 plasmid |
| Pgap2 Guide 6 F | caccgAGGGTCCCATCCCGATCCAG | Cloning of PGAP2 sgRNA into PX459 Cas9 plasmid |
| Pgap2 Guide 6 R | aaacCTGGATCGGGATGGGACCCTc | Cloning of PGAP2 sgRNA into PX459 Cas9 plasmid |
| Clpex KI sgRNA F | caccg CAGGTCTGACAAGATGTACC | Cloning of PGAP2 sgRNA into PX459 Cas9 plasmid to generate Clpex KI clones |
| Clpex KI sgRNA R | aaac GGTACATCTTGTCAGACCTGc | Cloning of PGAP2 sgRNA into PX459 Cas9 plasmid to generate Clpex KI clones |
| Clpex KI clone donor oligo | G\*G\*CTGCCTGGGGCCCTGACAGCATGCCACTCCATCCCCAGGTCTGACAAGgTGTACCAAGTCCCACTACCACTGGATCGGGATGGGACCCTGGTACGGCTC\*C\*G | Donor for homologous repair when used in combination with Clpex KI sgRNA to recapitulate the Clpex mutation in 293T cells |
| Kdm5 intron F | TGAAGCTTTTGGCTTTGAG | Genotyping of male/female mice using sexually dimprohic KDM5 intronic locus |
| Kdm5 intron R | CCGCTGCCAAATTCTTTGG | Genotyping of male/female mice using sexually dimprohic KDM5 intronic locus |
| mAlx3 in situ F | TGGAACCCTACCTCCCAGAG | Generation of Alx3 antisense RNA probe from plasmid |
| mAlx3 in situ R | ATTAACCCTCACTAAAGGTAGTCACCATCCGGAGAAGG | Generation of Alx3 antisense RNA probe from plasmid |
| Brachyury WMISH F | CCGGTGCTGAAGGTAAATGT | Generation of Tbxt antisense RNA probe from plasmid |
| Brachyury WMISH R | ATTAACCCTCACTAAAGGTGACCGGTGGTTCCTTAGAG | Generation of Tbxt antisense RNA probe from plasmid |
| mLHX8 WMISH F | TGTAAGCTGGAGGGAAAGGA | Generation of Lhx8 antisense RNA probe from plasmid |
| mLHX8 WMISH R | ATTAACCCTCACTAAAGGTTGGATGATTGACGTCTTGC | Generation of Lhx8 antisense RNA probe from plasmid |
| PigP WMISH F | GCAGGGTCTGCACTCACC | Generation of Pigp antisense RNA probe from plasmid |
| PigP WMISH R | ATTAACCCTCACTAAAGGGGTATTGAGTTCTTTGGCTCCT | Generation of Pigp antisense RNA probe from plasmid |
| PigU WMISH F | GCACTGTTGGATCTGGGAGT | Generation of PigU antisense RNA probe from plasmid |
| PigU WMISH R | ATTAACCCTCACTAAAGGAGCAGACGATGATGATGCAG | Generation of PigU antisense RNA probe from plasmid |
| PigX WMISH F | AAGCCCCCAACTACTTGTCC | Generation of PigX antisense RNA probe from plasmid |
| PigX WMISH R | ATTAACCCTCACTAAAGGTGGCCATATTTGAAAACAGC | Generation of PigX antisense RNA probe from plasmid |
| Pgap2 in situ probe primer F | TTATAAAAGCTTGCGGCCGCAGAATATTGTACCAGGTCCCACTGACA | Generation of Pgap2 antisense RNA probe from plasmid |
| Pgap2 in situ probe primer R | GCTCTAGAAATTAACCCTCACTAAAGGCAGCTCTTTGTTCCCGAAGT | Generation of Pgap2 antisense RNA probe from plasmid |
| Pgap2 Sanger F | TGGCAGAGAGTTGCAGAGAA | For Sanger Sequencing of Clpex locus in Clpex line |
| Pgap2 Sanger R | AGCCCATGAGAACTGGCTAA | For Sanger Sequencing of Clpex locus in Clpex line |
