# Supplementary figures and images for: Glycosylphosphatidylinositol biosynthesis and remodeling are required for neural tube closure, heart development, and cranial neural crest cell survival
Source: eLife. 2019 Jun 24;8:e45248. doi: 10.7554/eLife.45248 (PMC6611694; doi:10.7554/eLife.45248)

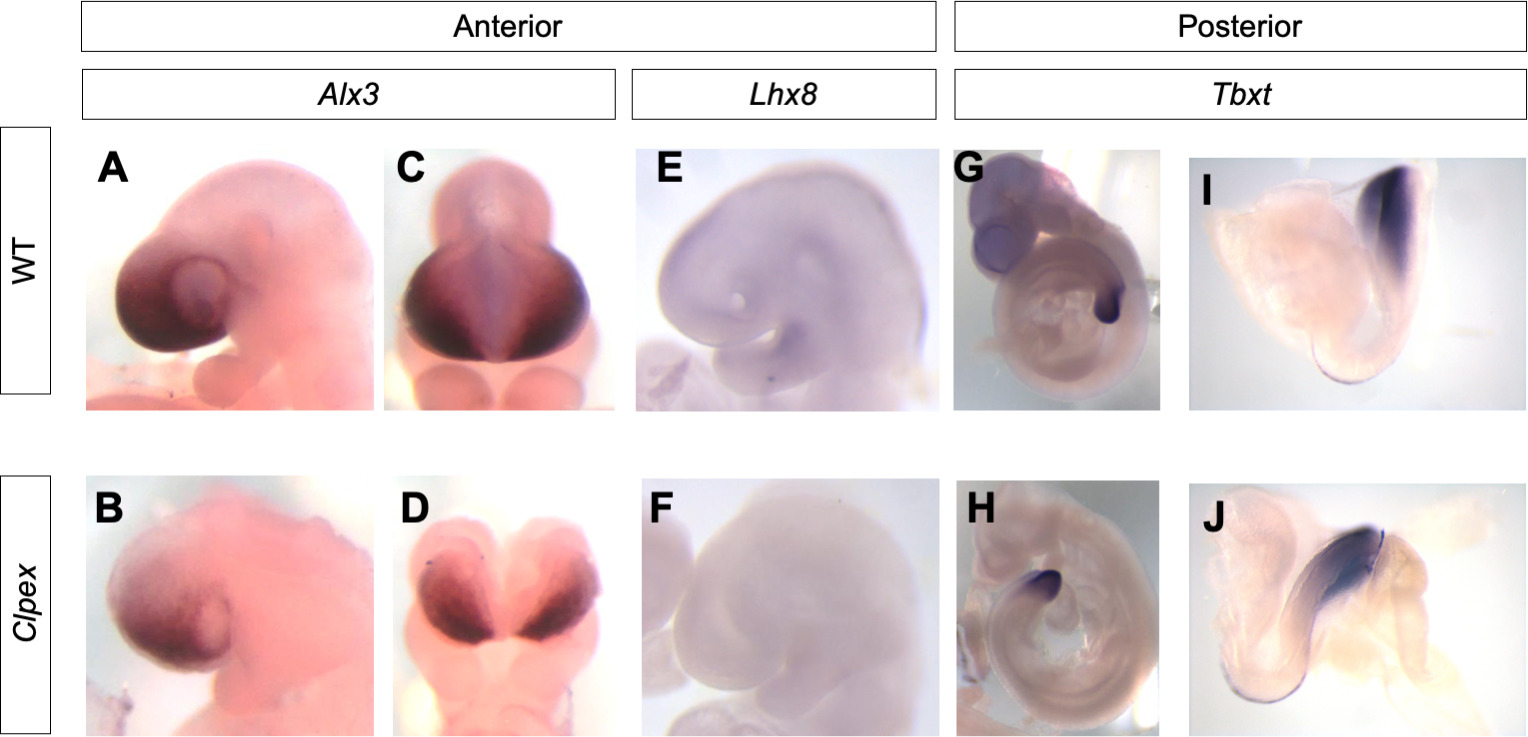

Supplement: Supplementary file 3. — E9.5 WT (A,C) and Clpex mutant (B, D) RNA in situ hybridization with α-sense Alx3 probe, an anterior pattering gene. E9.5 WT (E) and Clpex mutant (F) RNA in situ hybridization with α-sense Lhx8 probe, an anterior patterning gene. E9.5 WT (G) and Clpex mutant (H) RNA in situ hybridization with α-sense Tbxt (Brachyury) probe, a posterior patterning gene. E8.5 WT (I) and Clpex mutant (J) RNA in situ hybridization with α-sense Tbxt (Brachyury) probe, a posterior patterning gene. [file elife-45248-supp3.jpg]
