# Supplementary material for: Glycosylphosphatidylinositol biosynthesis and remodeling are required for neural tube closure, heart development, and cranial neural crest cell survival
Source: eLife. 2019 Jun 24;8:e45248. doi: 10.7554/eLife.45248 (PMC6611694; doi:10.7554/eLife.45248)
Supplement: Transparent reporting form [file elife-45248-transrepform.docx]

***eLife’s* transparent reporting form**

We encourage authors to provide detailed information *within their submission* to facilitate the interpretation and replication of experiments. Authors can upload supporting documentation to indicate the use of appropriate reporting guidelines for health-related research (see [EQUATOR Network](http://www.equator-network.org/%20)), life science research (see the [BioSharing Information Resource](https://biosharing.org/)), or the [ARRIVE guidelines](http://www.plosbiology.org/article/info:doi/10.1371/journal.pbio.1000412) for reporting work involving animal research. Where applicable, authors should refer to any relevant reporting standards documents in this form.

If you have any questions, please consult our Journal Policies and/or contact us: [editorial@elifesciences.org](mailto:editorial@elifesciences.org).

**Sample-size estimation**

- You should state whether an appropriate sample size was computed when the study was being designed
- You should state the statistical method of sample size computation and any required assumptions
- If no explicit power analysis was used, you should describe how you decided what sample (replicate) size (number) to use

Please outline where this information can be found within the submission (e.g., sections or figure legends), or explain why this information doesn’t apply to your submission:

We did not perform a power analysis before each experiment. We set up the experiments generally following the developmental biology conventions that any qualitative result should be seen in three replicates in order to be believed and reported. We met or exceeded this expectation in all experiments with two noted exceptions listed in the replicates section where only two mutants were examined. FLAER and immunocytochemistry experiments were repeated in at least 3 technical replicates as is convention.

**Replicates**

- You should report how often each experiment was performed
- You should include a definition of biological versus technical replication
- The data obtained should be provided and sufficient information should be provided to indicate the number of independent biological and/or technical replicates
- If you encountered any outliers, you should describe how these were handled
- Criteria for exclusion/inclusion of data should be clearly stated
- High-throughput sequence data should be uploaded before submission, with a private link for reviewers provided (these are available from both GEO and ArrayExpress)

Please outline where this information can be found within the submission (e.g., sections or figure legends), or explain why this information doesn’t apply to your submission:

Biological replicates were performed from multiple *Clpex* mutants. Technical replicates were performed by analyzing the same cell lines for flow cytometry and immunocytochemistry staining over multiple experiments.

In general, the number of embryos analyzed and with a given phenotype is documented in each figure where relevant.

Figure 1 A-H. N=70 *Clpex* mutants were collected from over 10 litters for phenotypic analysis

Figure 1 I-L. N=5 *Clpex* mutants and 5 WT embryos were analyzed using skeletal preparation techniques across 3 litters

Figure 1M-P. N=12 *Clpex* mutants and 18 WT embryos were analyzed using skeletal preparation across 3 litters.

Figure 1 Q. Initial mapping was done with 4 mutants and subsequently the interval was refined with at least 36 mutant embryos.

Figure 1 R. N=3 *Clpex* and 3 WT embryos were Sanger sequenced

Table 1. N=3 *Clpex* embryos were sent for whole exome sequencing

Figure 2. N= 8 *Clpex*/null mutants were compared to 70 *Clpex/Clpex* mutants (embryos with each phenotype are displayed in Fig.2I)

Figure 3. N= 21 litters of *Pgap2*-LacZ+ embryos were analyzed at various developmental stages and data in Figure 3 are representative of expression seen in at least two embryos or sections

Figure 4A-B. N=3 biological replicates from 4 independently generated *Clpex/Clpex* and 4 WT MEF cell lines, each dot represents the Mean Fluorescence Intensity (MFI) for one experiment.

Figure 4C-D. N=3 technical replicates of FLAER staining in each cell line indicated, each dot represents the Mean Fluorescence Intensity (MFI) for one experiment.

Figure 5 A-I. N=3 technical replicates with FOLR1 staining in WT, PIGA^-/-^, and PGAP2^-/-^ cell lines.

Figure 5J. N=8-15 60x z-stack sections analyzed per genotype, with the Pearson coefficient from each image represented by one dot.

Figure 5K-L. Representative image from N=3 technical replicates of anti-myc western blot in WT and PIGA^-/-^ and PGAP2^-/-^ cell lines.

Figure 6A-F. Representative image from N= 2 *Clpex/Clpex*;*Wnt1*-Cre R26R embryos at age e9.5 and 5 e11.5 mutants for lineage trace experiments.

Figure 6G-P. Representative images of N=2 or more sections from 3 WT and 6 *Clpex* E9.5 mutants for analysis of CC3+ cells

Figure 6Q. N=2 or more sections from 3 WT and 6 *Clpex* E9.5 mutants for analysis of CC3+ cells with each dot representative of one image in which a Region of Interest was selected in the first branchial arch and CC3+ spots counted and AP2+ spots counted. Each dot represents the CC3+ spots divided by the AP2+ spots for one image taken at 20x magnification.

Figure 7. Number of embryos for each given phenotype are displayed in Fig. 6D.

Table 2. N=5 WT and 5 *Clpex* mutant e9.5 embryos were pooled and analyzed for RNA sequencing

Figure 8A-F. N= 3 biological replicates of the *Piga* α-sense probe and 2 replicates of *Piga* sense probe staining

Figure 8G-H. N=4 WT and 3 Mutant cell lines stained in two separate biological replicates. Each dot represents the MFI from one experiment.

Figure 9A-L. N= 6 mosaic cKOs and 7 hemizygous cKOs were analyzed phenotypically

Figure 9M-R. N=5 hemizgous cKOs were analyzed for skeletal preparation

Figure S2. N= 21 litters of *Pgap2*-LacZ+ embryos were analyzed at various developmental stages and data in Figure 3 are representative of expression seen in at least two embryos or sections

Figure S3. DNA from *PIGA*-/-, *PGAP2*-/-, and WT 293T cell lines were sent for Sanger Sequencing once and intermittently re-genotyped by PCR to confirm the respective deletions were maintained. Three independent clones of the *Clpex* KI line were sent for Sanger Sequencing.

Figure S4. N=3 litters, 12 *Clpex* mutants were analyzed with the Toluidine blue barrier assay.

Figure S5. N=2 WT embryos fed the control diet, 1 image/mouse was analyzed. N=2 *Clpex* mutants fed the control diet, 3 or more images/mouse were analyzed. N=3 WT embryos fed the folinic acid diet, 1 image/mouse was analyzed. N=7 *Clpex* mutants fed the folinic acid diet, 2 or more images/mouse were analyzed and quantified. Each dot represents the CC3+ spots/AP2+ spots per image.

Figure S6. N=2 or more *Clpex* mutants were analyzed for each probe displayed.

Figure S7. N=2 or more biological replicates of each *Pig* αsense probe and at least 2 WT embryos stained with the respective sense control.

**Statistical reporting**

- Statistical analysis methods should be described and justified
- Raw data should be presented in figures whenever informative to do so (typically when N per group is less than 10)
- For each experiment, you should identify the statistical tests used, exact values of N, definitions of center, methods of multiple test correction, and dispersion and precision measures (e.g., mean, median, SD, SEM, confidence intervals; and, for the major substantive results, a measure of effect size (e.g., Pearson's r, Cohen's d)
- Report exact p-values wherever possible alongside the summary statistics and 95% confidence intervals. These should be reported for all key questions and not only when the p-value is less than 0.05.

Please outline where this information can be found within the submission (e.g., sections or figure legends), or explain why this information doesn’t apply to your submission:

All statistical tests are stated in the Statistical analysis section of the Materials and Methods. Statistics were generated using unpaired, two-tailed student’s t-test for comparisons between two groups, one way ANOVA with the p-value reported using Tukey’s multiple comparison’s test for more than two groups, or z-test of proportions for diet phenotype comparisons. P values were reported in each figure and added to the text. Values found not significant are reported as “n.s”. One-way ANOVA was chosen to determine the p-value for differences in FLAER staining between *Clpex* KI Clones, *PGAP2^-/-^*, and *PIGA ^-/-^* clones (Fig 4). One-way ANOVA was also chosen to analyze the statistical difference between Pearson’s coefficient between WT, *PGAP2^-/-^*, and *PIGA ^-/-^* clones (Fig. 5). We chose one-way ANOVA as we sought to determine differences in the mean of three or more unrelated groups The phenotypic results under various diet conditions were analyzed using the z-test of proportions (Fig.6). We chose z-test because there were multiple treatment groups with multiple phenotypes/group and we wanted to analyze the proportion of mutants with each phenotype under each treatment regimen. P values for z-test are available in Fig. 6D.

(For large datasets, or papers with a very large number of statistical tests, you may upload a single table file with tests, Ns, etc., with reference to sections in the manuscript.)

**Group allocation**

- Indicate how samples were allocated into experimental groups (in the case of clinical studies, please specify allocation to treatment method); if randomization was used, please also state if restricted randomization was applied
- Indicate if masking was used during group allocation, data collection and/or data analysis

Please outline where this information can be found within the submission (e.g., sections or figure legends), or explain why this information doesn’t apply to your submission:

Masking was not used in data collection or analysis. Phenotypic differences between WT and *Clpex* mutant precluded blind acquisition of most of the data.

**Additional data files (“source data”)**

- We encourage you to upload relevant additional data files, such as numerical data that are represented as a graph in a figure, or as a summary table
- Where provided, these should be in the most useful format, and they can be uploaded as “Source data” files linked to a main figure or table
- Include model definition files including the full list of parameters used
- Include code used for data analysis (e.g., R, MatLab)
- Avoid stating that data files are “available upon request”

Please indicate the figures or tables for which source data files have been provided:

Figure 1, 4, 5, 6, 8
